# Aspirin associated with risk reduction of secondary primary cancer for patients with head and neck cancer: A population-based analysis

**DOI:** 10.1371/journal.pone.0199014

**Published:** 2018-08-22

**Authors:** Yu-Shan Lin, Chih-Ching Yeh, Shiang-Fu Huang, Yi-Sheng Chou, Li-Tang Kuo, Fung-Chang Sung, Chih-Hsin Muo, Chien-Tien Su, Fu-Hsiung Su

**Affiliations:** 1 Department of Family Medicine, Taipei Medical University Hospital, Taipei, Taiwan; 2 School of Public Health, College of Public Health, Taipei Medical University, Taipei, Taiwan; 3 Department of Public Health, China Medical University, Taichung, Taiwan; 4 Department of Otolaryngology, Head and Neck Surgery, Chang Gung Memorial Hospital, Linkou, Taiwan; 5 Department of Public Health, Chang Gung University, Tao-Yuan, Taiwan; 6 Division of Hematology and Oncology, Department of Medicine, Taipei City Hospital, Renai Branch, Taipei, Taiwan; 7 School of Medicine, National Yang-Ming University, Taipei, Taiwan; 8 Division of Cardiology, Department of Internal Medicine, Chang Gung Memorial Hospital, Keelung, Taiwan; 9 Department of Health Services Administration, College of Public Health, China Medical University, Taichung, Taiwan; 10 Management Office for Health Data, China Medical University Hospital, Taichung, Taiwan; 11 School of Medicine, College of Medicine, Fu Jen Catholic University, New Taipei City, Taiwan; 12 Division of Family Medicine, Department of Community Medicine and Long Term Care, Fu Jen Catholic University Hospital, Fu Jen Catholic University, New Taipei City, Taiwan; 13 School of Medicine, Flinders University, Bedford Park, Australia; 14 Department of Family Medicine, Cardinal Tien Hospital, Fu Jen Catholic University, New Taipei City, Taiwan; University of Toronto, CANADA

## Abstract

As reported by the Taiwan Cancer Registry in 2013 squamous cell carcinoma of head and neck cancer (HNSCC) was the sixth most frequently diagnosed cancer and the 5^th^ most common cause of cancer related death and its incidence and mortality rate is still rising. The co-occurrence of HNSCC and secondary primary cancer (SPC) and the chemopreventive effect of aspirin on certain malignancies had been reported. Therefore we conducted this national study to investigate the use of aspirin associated with risk reduction of secondary primary cancer for patients with head and neck cancer in Taiwan. We searched the Registry for Catastrophic Illness in the National Health Insurance Research Database (NHIRD) for 18,234 patients (3,576 aspirin users and 14,667 non-aspirin users) diagnosed with HNSCC during 2000–2005. The SPC incidence density during follow-up in 2000–2011 was compared between the groups. For HNSCC patients, aspirin use after diagnosis was significantly associated with SPC risk reduction by 25% (adjusted HR, 0.75; 95% CI, 0.63–0.89; p = 0.001) after multivariate analysis. In the subgroup analysis, we found that esophageal cancer and stomach cancer incidence were significantly reduced after aspirin use (adjusted HR, 0.60; 95% CI, 0.41–0.90; p = 0.01 for esophageal cancer; adjusted HR, 0.27; 95% CI, 0.08–0.87; p = 0.03 for stomach cancer). Aspirin use for 1–3 years was associated with SPC risk reduction by 35% (adjusted HR, 0.65; 95% CI, 0.49–0.87; p = 0.003). SPC risk reduction extended continuously for more than 3 years of follow up (adjusted HR, 0.72; 95% CI, 0.53–0.98; p = 0.030). Our data shows aspirin use was associated with reduced SPC incidence for HNSCC patients, attributed mainly to reduced risk of esophageal and stomach cancer.

## Introduction

The cancer preventive effect of aspirin is inconclusive and debatable so far. Yet several rational biological mechanisms of anticancer effects of aspirin have been discussed in numerous studies. Which comprised inducing cell apoptosis, and inhibition of cyclooxygenase enzymatic production of prostaglandins [1]. Based on overall review of literatures, most strong evidence of positive chemopreventive effect was derived from the use of aspirin in colorectal cancer, and regular aspirin use was associated with a 27% reduced risk of colorectal cancer in a large meta-analysis of 37,519 cases (Relative risk, 0.73; 95% CI, 0.67–0.79; p<0.001) [2]. Despite most meta-analysis and observational epidemiological research repeatedly demonstrated its benefit in prevention of colorectal cancer [2–4], nonetheless, the intervention of aspirin to prevent cancer in randomized control study often failed to consistently demonstrate clinical evidence of cancer chemoprevention of aspirin [5–7]. Several bias and confounding factors preclude reaching consistent conclusions in either case-control, cohort, or randomized control studies, such as publication bias, detection bias, recall bias, immortal time bias, the dose of aspirin, and heterogeneity of study design.

According to Taiwan Cancer Registry in 2013, squamous cell carcinoma of head and neck cancer (HNSCC) was the sixth most frequently diagnosed cancer and the 5^th^ most common cause of cancer related death. Around 7300 patients were diagnosed of HNSCC and 2200 patients died of the disease annually in Taiwan [8]. The incidence and mortality rate is still rising. There were 3 major etiopathogenesis for HNSCC in Asia including tobacco, alcohol and betel nut [9–10]. The infection of human papilloma virus (HPV) is also increasingly recognized as risk factor for development of HNSCC [11]. The HNSCC comprises of squamous cell carcinoma (SCC) located at the oral cavity, oropharynx, hypopharynx, and larynx. Since the prognosis and management of SCC of nasopharynx and sino-nasal tract is distinct from HNSCC of other area, they were beyond the scope of discussion in this paper. The main treatment of primary HNSCC is upfront surgery or alternatively, chemotherapy and radiotherapy if surgical options are not considered feasible. However, second primary cancer remains at high risk even after the cure of index HNSCC and the reported incidence of secondary primary cancer (SPC) in patient with HNSCC is 3–7% per year, which primarily involves head and neck, lung and esophagus [12].

Aspirin as a secondary prevention for cancers have been explored in numerous prior studies, where evidence have shown that aspirin use has improved colorectal cancer specific survival rate (CSS) and overall survival (OS) [13]. However to the best of our knowledge, there were only sparse epidemiological researches exploring the potential chemoprevention effect of aspirin on SPC particular HNSCC. For this reason, the goal of our investigation is to utilize a large population data base to find out if the use of aspirin reduces the risk of SPC in patients diagnosed of primary HNSCC in a both high incidence and prevalence area.

## Patients and methods

### Study design, patient population, and data collection

This study was approved by the Institutional Review Board of China Medical University and Hospital Research Ethics Committee (IRB approval number: CMU-REC-101-012). We use the National Health Insurance Research Database (NHIRD) to conduct this population-based retrospective cohort study. The NHIRD was generated from data from the National Health Insurance (NHI) program, which provides medical care for nearly all of the 23 million residents in Taiwan. The NHIRD gave permission to public access of medical claims for the insured beneficiaries [14–16].

**Fig 1:**
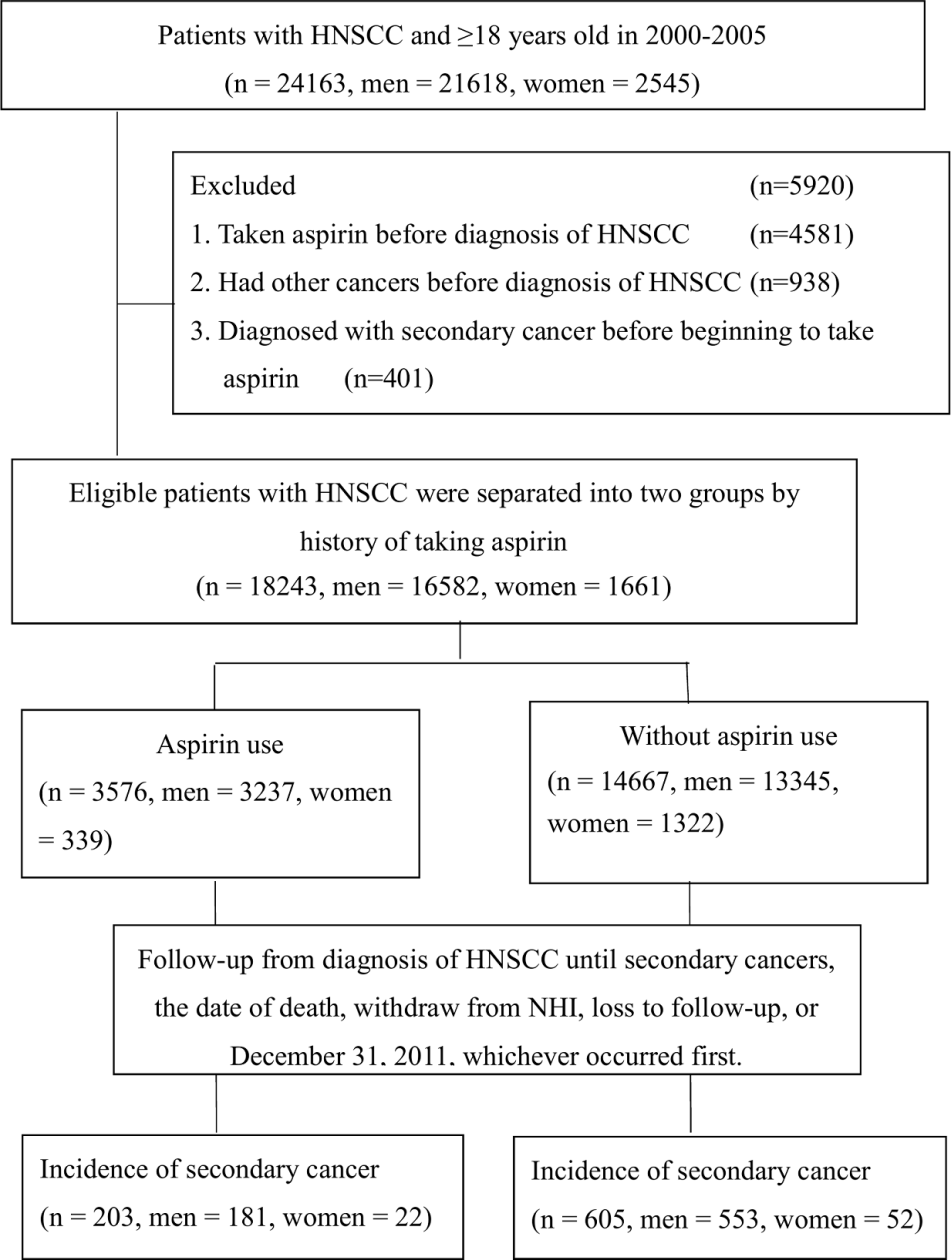
Study population selection. Patients with squamous cell carcinoma of head and neck cancer (HNSCC) were selected from the Registry for Catastrophic Illness Patient (RCIP) database in the National Health Insurance Research Database (NHIRD) in Taiwan.

Our research was in accordance with the Helsinki Declaration. All the individual identifiable information of patients was scrambled to ensure personal privacy. We used the longitudinal cohort of a randomly selected one million patients’ claim database based on the Registry for Catastrophic Illness Patient Database from the years of 2000 to 2011. The eligibility criteria included patients with diagnosis of squamous cell carcinoma of head and neck cancer (HNSCC) during the interval of 2000 to 2005, and age more than 18 years. Patients with diagnosis of HNSCC prior to the year of 2000, patients taking aspirin prior to diagnosis of HNSCC or after the diagnosis of second primary cancer (SPC) were excluded from our analysis. Patients with HNSCC were followed from the year of 2000 until December 31^st^, 2011 or censored due to death, withdraw from NHI or loss to follow-up. All the eligible patients were separated into two groups by history of taking aspirin into aspirin user and non-aspirin user. We identified 3,576 aspirin users and 14,667 non-aspirin users with HNSCC. We compared incidences of SPC after diagnosis of HNSCC during the interval of 2000 to December 31^st^, 2011 for aspirin users and on-aspirin users with HNSCC. (Fig 1)

### Criteria and definition

Patients with diagnosis of HNSCC was defined as ICD-9-M code 140–149, but nasopharyngeal carcinoma (ICD-9-M code 147), malignant neoplasm of nasal cavity (ICD-9-M code 160), cancer of frontal sinus (ICD-9-M code 148.1), cancer of sphenoidal sinus (ICD-9-M code 160.5), cancer of maxillary sinus (ICD-9-M code 160.2), and cancer of ethmoidal sinus (ICD-9-M code 160.3) were excluded. We defined aspirin users as those prescribed 50–150 mg of commercially available aspirin products for at least 6 consecutive months [17,18]. Patients taking aspirin were identified and extracted from the NHRI prescription data base by using the code N02BA01 (B01AC06) of the anatomic therapeutic chemical (ATC) classification system. The primary outcome was the incidence of SPC which was defined as head neck cancer (ICD-9-M code 140–149), esophageal cancer (ICD-9-M code 150), stomach cancer (ICD-9-M code 151), colorectal cancer (ICD-9-M code 153–154), liver cancer (ICD-9-M code 155), gallbladder and extrahepatic bile duct cancer (ICD-9-M code 156), pancreas cancer (ICD-9-M code 157), lung cancer (ICD-9-M code 162), melanoma (ICD-9-M code 172), skin cancer melanoma (ICD-9-M code 173),breast cancer (ICD-9-M code 174), melanoma (ICD-9-M code 172),uterus and corpus cancer (ICD-9-M code 179 and 182), cervical cancer (ICD-9-M code 180), ovary cancer (ICD-9-M code 183), prostate cancer (ICD-9-M code 185), bladder cancer (ICD-9-M code 188), kidney cancer (ICD-9-M code 189), brain cancer melanoma (ICD-9-M code 191), thyroid gland cancer (ICD-9-M code 193), non-Hodgkin’s lymphoma (ICD-9-M code 202), myeloma (ICD-9-M code 203) and leukemia (ICD-9-M code 204–208).

Statin is a class of drug that inhibits 3-hydroxy-3-methylglutaryl coenzyme A (HMG-CoA) reductase which is the rate-limiting enzyme in the synthesis of mevalonate [19,20]. This type of medication is designed to block HMG-CoA reductase in turn efficiently control hypercholesterolemia. Since HMG-CoA reductase is involved in cholesterol synthesis and growth control, statins may have chemopreventive activity against cancer [21]. The prescribed statin included atorvastatin (ATC code C10AA05), rosuvastatin (ATC code C10AA07), lovastatin (ATC code C10AA02), simvastatin (ATC code C10AA01), pravastatin (ATC code C10AA03), and fluvastatin (ATC code C10AA04). Coexisting medical disorders and associated comorbidities includes hypertension (ICD-9-CM code 401–405), diabetes mellitus (DM, ICD-9-CM code 250), coronary heart disease (CHD, ICD-9-CM code 410–414 or 429.2), heart failure (HF, ICD-9-CM code 428), atrial fibrillation (AF, ICD-9-CM code 427.31), chronic kidney disease (CKD, ICD-9-CM code 585) and hyperlipidemia (ICD-9-CM code 272).

### Statistical and survival analysis

Outcome measurement of incidence rate was defined as the number of incident event of diagnosis of SPC divided by the sum of the person-year of the at risk population during the interval of follow- up. Incidence curves were estimated using the Kaplan–Meier method and compared by log-rank tests. The secondary cancer free survival was defined from the date of diagnosis of HNSCC to the date of SPC, the last date of follow-up or December 31^st^, 2011 if no evidence of SPC or loss to follow-up. The associations between nominal data were analyzed using the chi-square test. The multivariate Cox proportional hazard model was used for adjusted hazard ratio and 95% confidence interval (95% CI) by variables of age, sex, urbanization, comorbidity, such as CHD, hypertension, DM, Af, HF, hyperlipidemia, CKD and associated medicine use of cyclooxygenase 2 (COX2) inhibitor, and statin. A *p* value less than 0.05 in two-sided test was considered as statistically significant. Statistics and survivals were examined by SAS statistical software (Version 9.4 for Windows; SAS Institute, Inc., Cary, NC, USA)

## Results

### Clinicopathological characteristics of patients with squamous cell carcinoma of head and neck cancer (HNSCC)

A total of 24,163 patient aged ≥ 18 years old were newly diagnosed of HNSCC during the interval of 2000–2005 in the longitudinal cohort of a randomly selected one million patients’ claim database based on the registry for catastrophic illness from the years of 2000 to 2011. Among them, 21,618 patients were men (89.5%) and 2,545 patients were women (10.5%). 4,581 patients who had taken aspirin prior to diagnosis of HNSCC were excluded. 938 patients had diagnosis of cancer prior to 2000 were excluded. 401 patients taking aspirin after the diagnosis of SPC were also excluded. 3,576 aspirin users were identified (3,237 men (90.5%); 339 women (9.5%)) and 14,667 non-aspirin users were identified (13,345 men (91%); 1322 women (9%)).

Next, we examined incidence of SPC during the interval of 2000–2011. We found 203 patients in the group of aspirin users (181 men and 22 women) and 605 patients in the group of non-aspirin users (553 men and 52 women). The top six distribution sites of SPC among HNSCC patients in our study were esophagus (24.1%), lung (19.2%), liver (13.6%), colorectum (7.7%), head and neck (3.7) and stomach (3.5%), respectively. In comparison with the use of aspirin, more esophagus cancer was observed in non-aspirin users (26.8% vs 16.3% p = 0.002) (S1 Table).

Table 1 demonstrated the demographics of patients with HNSCC. Compared to non-aspirin users, there were more patients who were older (52.9 versus 49.5 years old), inhabited in the non-urban area, and having comorbidity including CHD, hypertension, DM, Af, HF, hyperlipidemia and CKD, and taking medicines of COX2 inhibitor and statins. The median follow-up period for all HNSCC patients were 1.27 years (0.39–3.98 years) and 2.98 years (0.96–5.96 years) as well as 1.02 (0.34–3.42 years) for aspirin users and non-aspirin users respectively.

**Table 1:** Demographic characteristics and comorbidities of HNSCC in Taiwan (n = 18243).

	All		Aspirin user		Non-aspirin user		
Variable	n	(%)	n	(%)	n	(%)	p
Age, year							
Median (IQR)	50.21	(43.38~58.92)	52.92	(45.92~61.83)	49.57	(42.82~58.98)	<0.0001
18–30	417	2.29	50	1.40	367	2.50	<0.0001
31–40	2847	15.61	387	10.82	2460	16.77	
41–50	6373	34.93	1105	30.90	5268	35.92	
51–60	4791	26.26	1068	29.87	3723	25.38	
61–70	2495	13.68	650	18.18	1845	12.58	
≥71	1320	7.24	316	8.84	1004	6.85	
Gender							0.38
Male	16582	90.90	3237	90.52	13345	90.99	
Female	1661	9.10	339	9.48	1322	9.01	
Urbanization							0.003
Urban	9928	54.42	1861	52.04	8067	55.00	
Suburban	5808	31.84	1176	32.89	4632	31.58	
Rural	2507	13.74	539	15.07	1968	13.42	
Follow-up year, median (IQR)	1.27	(0.39~3.98)	2.98	(0.96~5.96)	1.02	(0.34~3.42)	<0.0001
**Comorbidities**							
Coronary heart disease	1268	6.95	719	20.11	549	3.74	<0.0001
Hypertension	4450	24.39	1624	45.41	2826	19.27	<0.0001
Diabetes	2612	14.32	902	25.22	1710	11.66	<0.0001
Atrial fibrillation	54	0.30	40	1.12	14	0.10	<0.0001
Heart failure	223	1.22	115	3.22	108	0.74	<0.0001
Hyperlipidemia	2311	12.67	856	23.94	1455	9.92	<0.0001
Chronic kidney disease	225	1.23	85	2.38	140	0.95	<0.0001
**Medicines**							
COX2	6355	34.84	1580	44.18	4775	32.56	<0.0001
Statins	1696	9.30	918	25.67	778	5.30	<0.0001

Abbreviations: COX2, cyclooxygenase 2; IQR, interquartile range

### Incidence of second primary cancer (SPC) in aspirin users and non-aspirin users

**Fig 2:**
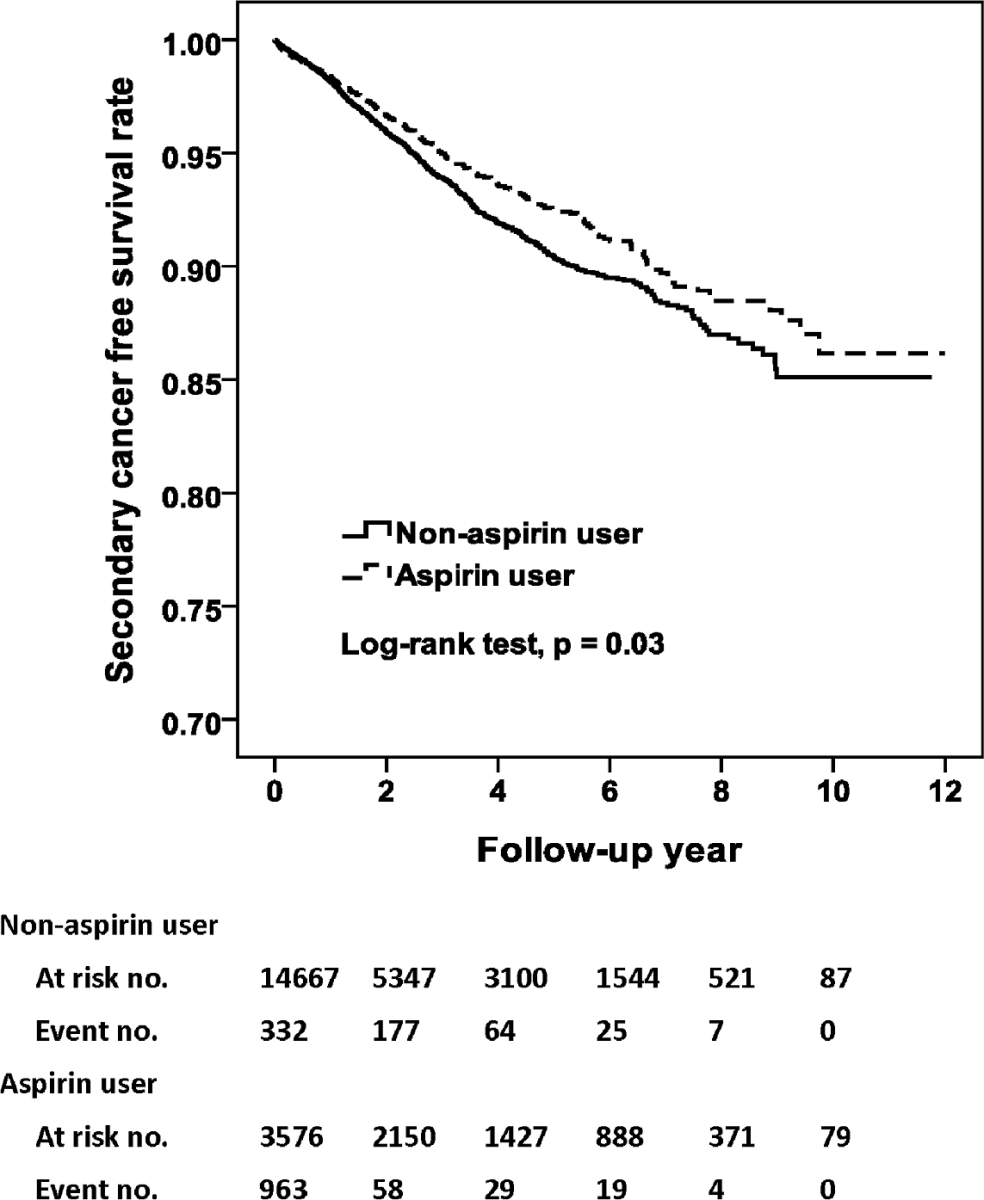
Secondary cancer free survival rates for primary HNSCC cohort with and without Aspirin intake.

**Fig 3:**
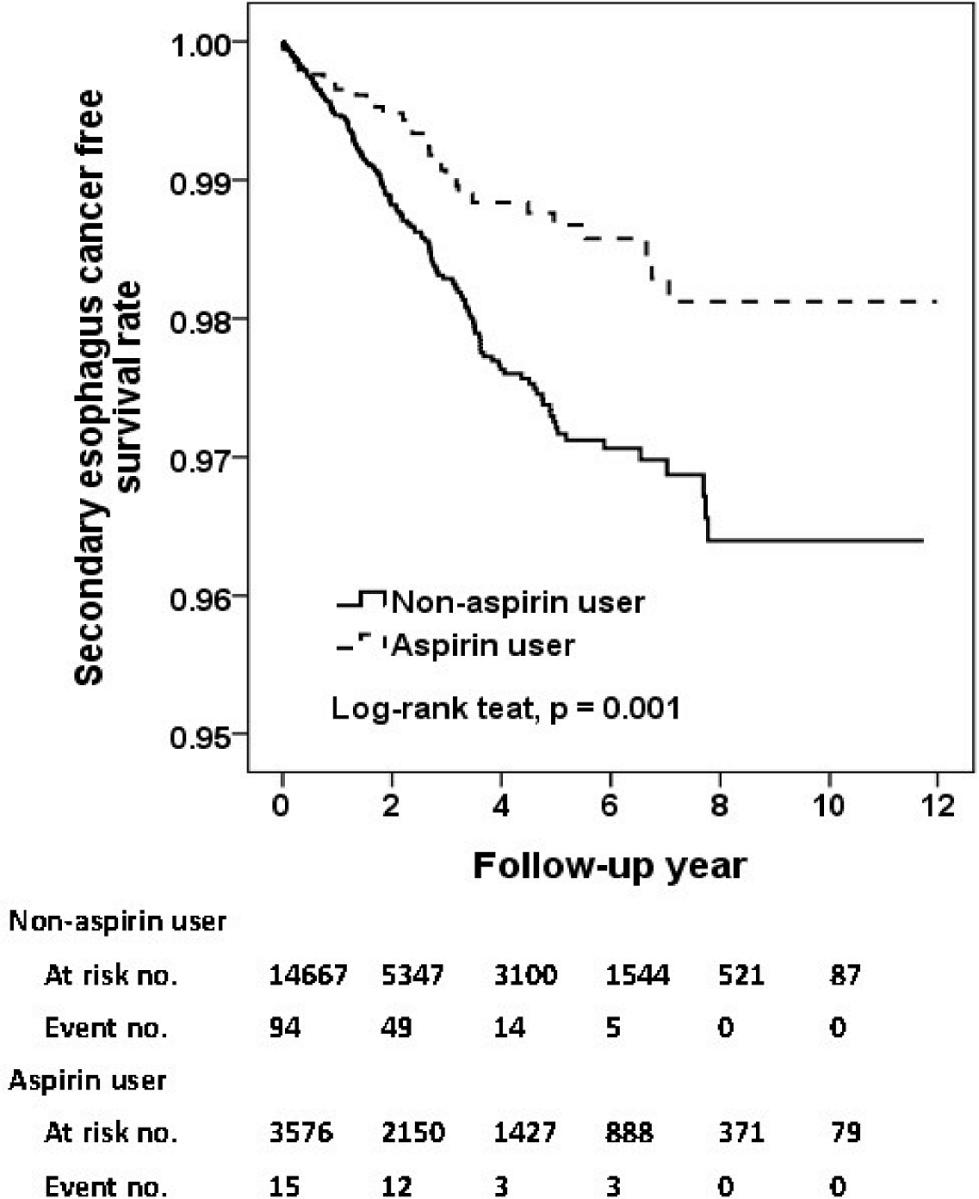
Secondary esophagus cancer free survival rates for primary HNSCC cohort with and without aspirin intake.

**Fig 4:**
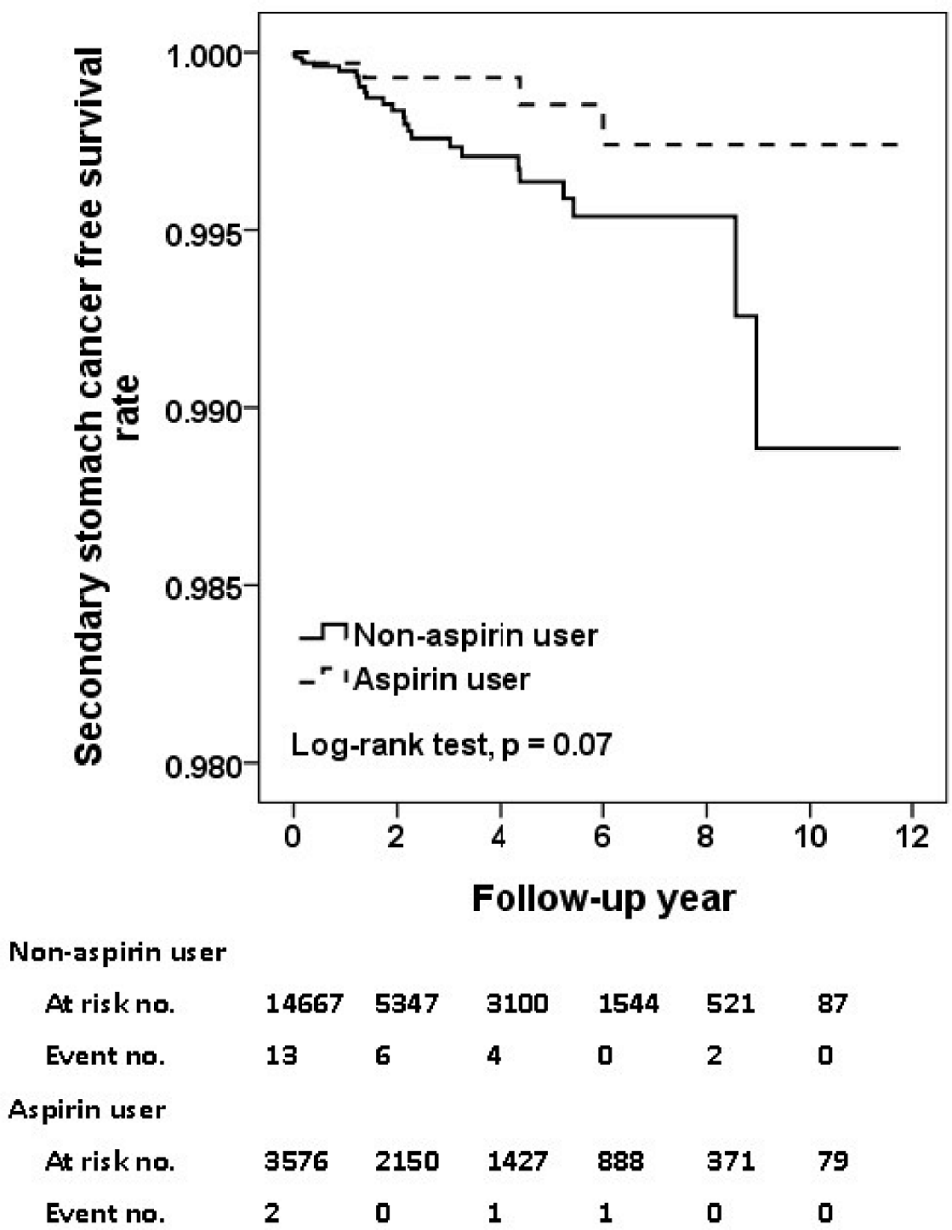
Secondary stomach cancer free survival rates for primary HNSCC cohort with and without aspirin intake.

In Table 2, the incidence rate of SPC was 15.6 and 19.0 per 1000 person-year for aspirin users and non-aspirin users, respectively. The crude HR of SPC was 0.84 for aspirin compared to non-aspirin users. After adjustment, the use of aspirin was associated with a 25%- reduction of risk of SPC (adjusted HR, 0.75; 95% CI, 0.63–0.89; p = 0.001). As shown in Fig 2, the aspirin users had a significantly superior free survival rate compared to non-aspirin users (p = 0.03, Fig 2). As demonstrated in Table 3, regarding the specific cancer subtypes, use of aspirin was significantly associated with reduced incidence of esophageal cancer (adjusted HR, 0.60; 95% CI, 0.41–0.90; p = 0.01; Fig 3), and stomach cancer (adjusted HR, 0.27; 95% CI, 0.08–0.87; p = 0.03; Fig 4).

**Table 2:** Incidence density of secondary primary cancer in aspirin users and non-aspirin users among HNSCC patients identified during 2000–2011.

Aspirin use	N	Event	PY^a^	Rate^b^	Crude HR(95% CI)	*P*	Adjusted HR (95% CI)^c^	*P*
No	14667	605	31796	19.03	1.00 (reference)		1.00 (reference)	
Yes	3576	203	13013	15.60	0.84 (0.72–0.99)	0.03	0.75 (0.63–0.89)	0.001

^a^ Person-year

^b^ Rate: Incidence rate per 1000 person-year

^c^ Adjusted for age, sex, urbanization, coronary heart disease, hypertension, diabetes, atrial fibrillation, heart failure, hyperlipidemia, chronic kidney disease, and medicines use of COX2, and statins.

**Table 3:** The incidence of major secondary primary cancer in aspirin users and non-aspirin users among HNSCC patients identified during 2000–2011.

	Aspirin user (N = 3576)		Non-aspirin user (N = 14667)			
Cancer Type	Event	Rate^a^	Event	Rate^a^	HR (95% CI)^b^	*P*
Head and neck cancer	9	0.69	21	0.66	1.44 (0.62–3.37)	0.39
Esophagus cancer	33	2.54	162	5.10	0.60 (0.41–0.90)	0.01
Stomach cancer	4	0.31	25	0.79	0.27 (0.08–0.87)	0.03
Colorectal cancer	16	1.23	46	1.45	0.59 (0.31–1.11)	0.10
Liver cancer	28	2.15	82	2.58	0.67 (0.42–1.08)	0.10
Lung cancer	37	2.84	118	3.71	0.71 (0.18–1.07)	0.10
Bladder cancer	6	0.46	9	0.28	0.83 (0.26–2.62)	0.83

^a^ Rate: Incidence rate per 1000 person-year

^b^ Adjusted for age, sex, urbanization, coronary heart disease, hypertension, diabetes, atrial fibrillation, heart failure, hyperlipidemia, chronic kidney disease, and medicines use of COX2, and statins.

### The incidence density of SPC stratified by durations of follow-up

To determine whether there was exposure-response correlation between use of aspirin and incidence of SPC, we stratify adjusted HR by duration of follow-up. As demonstrated in Table 4, it was of note that the use of aspirin was associated with a risk reduction of SPC by 35% during the follow-up of 1 to 3 after diagnosis of HNSCC (adjusted HR, 0.65; 95% CI,0.49–0.87; p = 0.003). The extent of risk reduction persisted over time if the patient with HNSCC received surveillance more than 3 years, (adjusted HR, 0.72; 95% CI, 0.53–0.98; p = 0.03).

**Table 4:** The incidence density of secondary primary cancer in Aspirin users and Non-aspirin users among HNSCC patients stratified by follow-up duration.

	Aspirin user			Non-aspirin user				
Follow-up year	N	Event	Rate^a^	N	Event	Rate^a^	HR (95% CI)^b^	*P*
≤ 1.0	3576	51	16.58	14667	188	18.48	0.96 (0.69–1.33)	0.79
1.1–3.0	2658	77	17.71	7423	243	22.27	0.65 (0.49–0.87)	0.003
>3.0	2150	75	12.99	5347	174	15.40	0.72 (0.53–0.98)	0.03

^a^ Rate: Incidence rate per 1000 person-year

^b^ Adjusted for age, sex, urbanization, coronary heart disease, hypertension, diabetes, atrial fibrillation, heart failure, hyperlipidemia, chronic kidney disease, and medicines use of COX2, statins, and NSAIDs.

## Discussion

Our population-based retrospective cohort study of more than 18,000 patient demonstrated that use of aspirin was significantly associated with a risk reduction of SPC by 25% (adjusted HR, 0.75; 95% CI, 0.63–0.89; p = 0.001) and better second primary cancer free survival. The most common sites of SPC among HNSCC patients in our study were esophagus, lung, liver, colorectum, head and neck and stomach in order. The cancer of lower incidence after the use aspirin includes esophageal cancer (adjusted HR, 0.60; 95% CI, 0.41–0.90; p = 0.01) and stomach cancer (adjusted HR, 0.27; 95% CI, 0.08–0.87; p = 0.03). The impact of aspirin grew more significantly prominent when SPC was diagnosed 1 to 3 years after the diagnosis of primary HNSCC and extended to more than three years of follow-up. As far as we know, while most of researches focused on the primary prevention of aspirin from risk of cancer, and a few of them found that aspirin improved survival and reduced distant metastasis after diagnosis of cancer [22], we are one of the first to demonstrate secondary chemoprevention effect of aspirin from risk of SPC. The reduced risk of SPC was mainly attributed to decreased esophageal and stomach cancer.

SPC developed in 27% after diagnosis of HNSCC and the accumulated incidence continued to rise to reach 61% at 25 years. One SPC occurred, the post-SPC survival was very dismal with a 5-year overall survival rate of 15% for second HNSCC patients [23].

The function of cyclooxygenase-2 (COX-2) inhibition has been recognized as major mechanism of chemoprevention for aspirin. Many growth factors and inflammatory cytokine known to promote cancer progression were produced, packaged and secreted by thrombocytes, such as platelet derived growth factor (PDGF), vascular endothelial growth factor (VEGF) and transforming growth factor-β (TGF-β) [24]. The inhibition of COX-2 enzymes in thrombocytes results in blockade of synthesis of prostaglandins (PGE) from arachidonic acid (AA).The downstream actions of PGE related with GF and cytokines plays essential roles in angiogenesis, cell proliferation and invasion. A platelet count of more than 400,000/μL was associated with higher mortality for patient with HNSCC (adjusted HR, 2.37; 95% CI 1.60–3.50) and the poor prognosis could be overcome by antiplatelet medications (adjusted HR, 0.42; 95% CI, 0.17–1.05) [25].

A recently published meta-analysis by Lanhua Tang, et al., found that long term usage of aspirin was associated a significant risk reduction of HNSCC (HR, 0.75; 95%CI, 0.65–0.85) but the findings could not be observed in the use of NSAID. (HR, 0.95; 95% CI, 0.81–1.11) [26]. It was noted that the evidence of beneficial impact of aspirin on risk of HNSCC predominantly originated form single paper by Jayaprakash et al., (HR, 0.75; 95% CI, 0.58–0.96) [27] but the other two papers by Macfarlane et al., did not support the hypothesis that aspirin prescription associated with risk reduction of HNSCC (adjusted odds ratio (OR), 0.9; 95% CI, 0.7–1.1; adjust OR 0.78; 95% CI, 0.58–1.05; respectively) [28,29]. Other 4 population based studies also demonstrated no protective benefit of aspirin on risk of HSNCC [30–33]. However, since a large number of our patients with HNSCC were at a high risk for SPC, it was of more power to detect the benefit of aspirin than the previous studies mentioned.

Interestingly enough, Macfarlane et al. also found that use of aspirin after diagnosis of HSNCC improved survival (HR, 0.56; 95% CI 0.44–0.71) [28,29]. In addition to HNSCC, a large meta-analysis of eight randomized trials of aspirin versus no aspirin, allocation to aspirin improved cancer specific survival (pooled odds ratio, 0.79, 95% CI, 0.68–0.92, p = 0.003), and the benefit was latent until a delay of 5 years [34]. Consistently shown in our study, the impact of aspirin increased over time (Table 4) and the risk reduction was most evident after follow-up of 1 to 3 years. As for the timeline where the onset of chemoprevention for aspirin might be most clinically apparent is similar to previous literature released [34]. The beneficial effect of aspirin might be partially attributed to reduced risk of distant cancer metastasis at relapse (all cancer, HR, 0.64, 95% CI, 0.48–0.84; adenocarcinoma, HR, 0.54, 95% CI< 0.38–0.77) [35].

There were several limitations in the design of our studies. First, as did retrospective population-based study have, lack of information regarding risk factors for HNSCC, stomach cancer, and esophageal cancer, such as consumptions of alcohol, cigarettes, and betel nut, HPV infection, and helicobacter pylori infection, our study result might be biased since the risk of SPC were not adjusted by the presence of these risk factors. In addition, while cancer histology (squamous cell carcinoma vs. adenocarcinoma), cancer stage and cancer treatment may have clinical impact on our result, it was not feasible to acquire these data in NHIRD. Furthermore, the risk of SPC was adjusted by use of COX-2 inhibitor but not NSAID since no information of NSAID were available. Compared to aspirin, the role of NSAID for patients with HNSCC was less conclusive [35], it was possible that the risk of SPC may be affected by the use of NSAID. Moreover, there were unbalanced distributions of clinical features between two groups of aspirin and non-aspirin users. Since the majority of patients with HNSCC were predominantly male around 90%, the chemoprevention effect of aspirin for woman should be carefully interpreted with caution and we could not examine SPC of breast and gynecologic cancer, such as ovary, endometrial and cervical cancer. Finally, there were no data of side effects, such as gastrointestinal bleeding, and stomach upsets collected for aspirin, so we cannot assess the balance of risk and benefit of aspirin.

Second, as we cannot retrieve the survival status, we cannot demonstrate the survival benefits for aspirin users, if present. However, as discussed previously, there have been many papers reporting survival benefit for aspirin after diagnosis of HNSCC [34,35]. Third, although the definition of aspirin use was a prescription of 50–150 mg of commercially available aspirin products for at least 6 consecutive months. We were not confirmed that all the patients had full compliance with the prescriptions. Besides, undetected over-the-counter aspirin would have us underestimate the benefit of aspirin. Moreover, it warrants further prospectively conducted randomized control study to validate the optimal dose, duration, and frequency (for example, every day vs. every other day) of aspirin to maximize the chemoprevention effect.

Fourth, since aspirin users had a significantly more risk factors for cardiovascular disease (CVD), they at higher risk to die of CVD. It is of great possibility that they were not surviving long enough to be diagnosed of SPC before mortality due to CVD. In such circumstances, it require a competing risk analysis for further clarification but as we previously discussed, it was not feasible for us not having survival status.

Fifth, since the duration of follow-up was not long enough to detect long term impact of aspirin, the clinical benefit of aspirin may be of greater degree than we reported if the use of aspirin was beyond 5–10 years [36]. It may also affect our sensitivity analysis (Table 4). Besides, we did not use ‘ever vs. never’ for the definition of aspirin use to avoid immortal time bias.

The strength of our study includes that the sample size was large enough to show statistical significance, and the patients were much likely to be representative of the general populations with HNSCC since nearly all the patients were registered in the data base.

In conclusion, aspirin use was associated with a significant risk reduction of SPC for patients with HNSCC, specifically on risk of stomach and esophageal cancer in this large population cohort. Given the high incidence of SPC and poor prognosis once occurred, more well-designed researche with a longer duration of use of aspirin and surveillance should address the observations and risk-benefit analysis prospectively in the future.

## Supporting information

S1 Table The distribution of secondary primary cancer in aspirin users and non-aspirin users among HNSCC patients identified during 2000–2011. (PDF)
