# Supplementary material for: Aspirin associated with risk reduction of secondary primary cancer for patients with head and neck cancer: A population-based analysis
Source: PLoS One. 2018 Aug 22;13(8):e0199014. doi: 10.1371/journal.pone.0199014 (PMC6104934; doi:10.1371/journal.pone.0199014)
Supplement: S1 Table — (PDF) [file pone.0199014.s001.pdf]

S1 Table The distribution of secondary primary cancer in aspirin users and non-aspirin users among HNSCC patients identified during 2000–2011

	All		Aspirin user		Non-aspirin user		
	N	(%)	N	%	N	%	p
Overall	808	100.00	203	100.00	605	100.00	
Brain cancer (191)	3	0.37	0	0.00	3	0.50	0.58
Head and neck cancer (140-149)	30	3.71	9	4.43	21	3.47	0.53
Thyroid gland cancer (193)	7	0.87	2	0.99	5	0.83	1.00
Esophagus cancer (150)	195	24.13	33	16.26	162	26.78	0.002
Stomach cancer (151)	29	3.59	4	1.97	25	4.13	0.19
Colorectal cancer (153, and 154)	62	7.67	16	7.88	46	7.60	0.90
Liver cancer (155)	110	13.61	28	13.79	82	13.55	0.93
Gallbladder and extra hepatic bile ducts (156)	6	0.74	2	0.99	4	0.66	0.64
Pancreas cancer (157)	10	1.24	5	2.46	5	0.83	0.13
Lung cancer (162)	155	19.18	37	18.23	118	19.50	0.69
Kidney cancer (189)	13	1.61	3	1.48	10	1.65	1.00
Bladder cancer (188)	15	1.86	6	2.96	9	1.49	0.18
Uterus and corpus cancer (women only) (179, and 182)	2	0.25	1	0.49	1	0.17	0.44
Cervical cancer (women only) (180)	2	0.25	1	0.49	1	0.17	0.44
Ovary cancer (women only) (183)	1	0.12	0	0.00	1	0.17	1.00
Prostate cancer (men only) (185)	14	1.73	5	2.46	9	1.49	0.36
Breast cancer (women only) (174)	12	1.49	6	2.96	6	0.99	0.045
Melanoma (172)	2	0.25	0	0.00	2	0.33	1.00
Skin cancer (173)	11	1.36	6	2.96	5	0.83	0.02
Non-Hodgkin's lymphoma (202)	16	1.98	2	0.99	14	2.31	0.38
Myeloma (203)	0	0.00	0	0.00	0	0.00	
Leukemia (204-208)	9	1.11	5	2.46	4	0.66	0.049
